# AMPKα phosphatase Ppm1E upregulation in human gastric cancer is required for cell proliferation

**DOI:** 10.18632/oncotarget.16126

**Published:** 2017-03-11

**Authors:** Min-Bin Chen, Yuan-Yuan Liu, Li-Bo Cheng, Jian-Wei Lu, Ping Zeng, Pei-Hua Lu

**Affiliations:** ^1^ Department of Radiotherapy and Oncology, Kunshan First People's Hospital Affiliated to Jiangsu University, Kunshan, China; ^2^ Department of Ophthalmology, Wuxi Second Hospital, Nanjing Medical University, Wu'xi, China; ^3^ Department of Oncology, Jiangsu Cancer Hospital Affiliated to Nanjing Medical University, Nanjing, China; ^4^ Department of Radiotherapy and Oncology, Wuxi People's Hospital Affiliated to Nanjing Medical University, Wuxi, China

**Keywords:** gastric cancer, AMPKα, Ppm1E, mTOR, miR-135b-5p

## Abstract

Activation of AMP-activated protein kinase (AMPK) is a valuable anti-cancer strategy. In the current study, we tested expression and potential function of Ca^2+^/calmodulin-dependent protein kinase phosphatase (Ppm1E), an AMPKα phosphatase, in human gastric cancers. Ppm1E expression was elevated in human gastric cancer tissues (*vs*. normal tissues), which was correlated with AMPK (p-AMPKα, Thr-172) dephosphorylation and mTOR complex 1 (mTORC1) activation. Ppm1E upregulation, AMPK inhibition and mTORC1 activation were also observed in human gastric cancer cell lines (AGS, HGC-27, and SNU601). Intriguingly, Ppm1E knockdown by shRNA induced AMPK activation, mTORC1 inactivation, and proliferation inhibition in AGS cells. On the other hand, forced over-expression of Ppm1E induced further AMPK inhibition and mTORC1 activation to enhance AGS cell proliferation. Remarkably, microRNA-135b-5p (“miR-135b-5p”), an anti-Ppm1E microRNA, was downregulated in both human gastric cancer tissues and cells. Reversely, miR-135b-5p exogenous expression caused Ppm1E depletion, AMPK activation, and AGC cell proliferation inhibition. Together, Ppm1E upregulation in human gastric cancer is important for cell proliferation, possible via regulating AMPK-mTOR signaling.

## INTRODUCTION

Gastric cancer has long been a major health threat [1]. Over the past decades, significant progress has been achieved in pathological mechanism research and therapeutic strategies for gastric cancer. Yet the prognosis has not been dramatically improved [2–4]. The five-year overall survival for those with advanced or recurrent metastatic gastric cancer is extremely poor [2–4]. Further, the incidence of this devastating disease has been rising in China [5] and other Eastern countries [1, 6]. The applications of the conventional cytotoxic drugs and newly molecular-targeted agents are not satisfactory in cancers with widespread pre-existing and/or acquired resistance [3, 4, 7].

AMP-activated protein kinase (AMPK) is the well-established master regulator of cellular energy metabolism [8, 9]. Existing literatures have implied that AMPK is also important for the regulation of cell survival and death (see review [8–10]). Our group [11–15] and others have indicated that AMPK activation could also promote cancer cell death via regulating the downstream targeting proteins. For example, in various cancer cells, forced-activation of AMPK, either pharmacologically and genetically, could induce p53 activation [16–18] and mammalian target of rapamycin (mTOR) complex 1 (mTORC1) inhibition [19], as well as autophagy induction [20, 21] and oncogenic protein degradation [22]. Many traditional cytotoxic chemo-drugs and natural compounds could provoke AMPK-dependent death pathway [10, 16, 23–32] in cancer cells.

Very recent research efforts have characterized Ca^2+^/calmodulin-dependent protein kinase phosphatase (Ppm1E) as a novel and vital AMPKα phosphatase [33–35]. On the other hand, Ppm1E silence or mutation could be a novel strategy to induce AMPKα1 phosphorylation and activation [33–35]. In this study, we show that Ppm1E, the AMPKα phosphatase, is significantly upregulated in human gastric cancer tissues and cell lines, which is possibly important for shutting down AMPK, thus promoting cancer cell proliferation.

## RESULTS

### Ppm1E upregulation in human gastric cancer tissues

**Figure 1:**
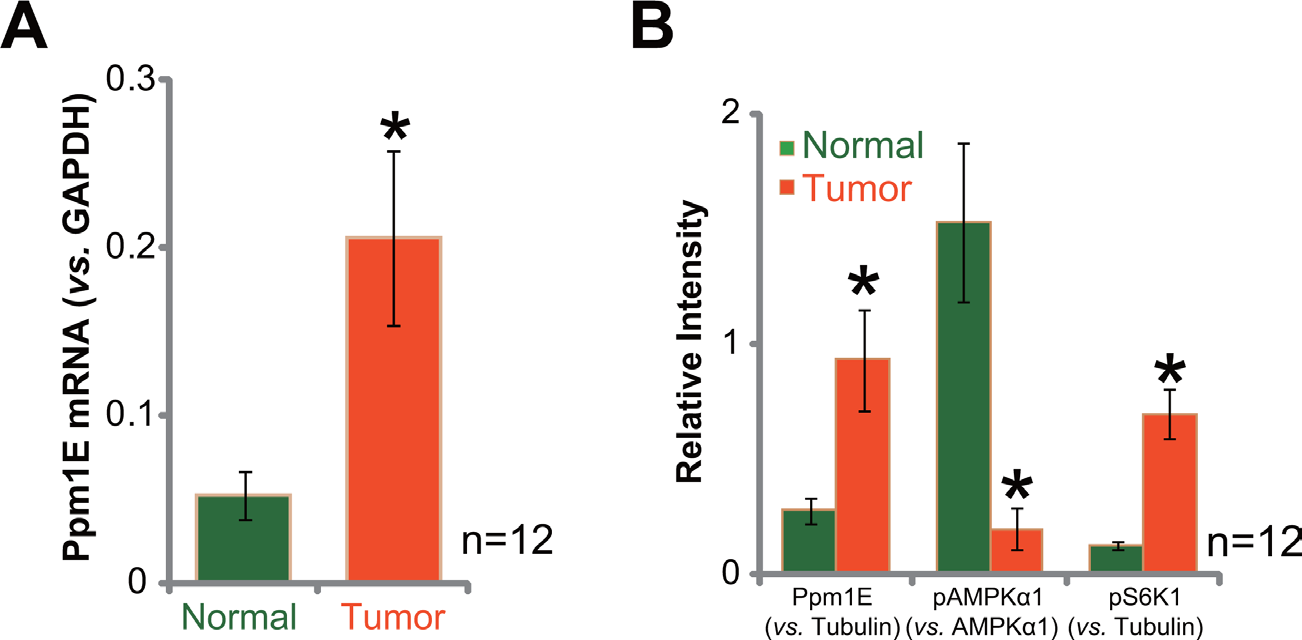
Ppm1E upregulation in human gastric cancer tissues The fresh human gastric cancer tissues (“Tumor”, *n* = 12) and the surrounding normal gastric tissues (“Normal”) were lysed; Expressions of Ppm1E mRNA (**A**, qRT-PCR assay) and listed proteins (**B**, Western blotting assay, Data were quantified) were tested. *p < 0.05 *vs*. “Normal”.

First, we tested Ppm1E expression in human gastric cancer tissues, and compared with the surrounding normal gastric tissues. A total of twelve (12) paired of fresh tissue specimens were analyzed. Quantitative real-time PCR assay (qRT-PCR assay) results in Figure 1A demonstrated that *Ppm1E* mRNA level was clearly elevated in gastric cancer tissues (“Tumor”), as compared that in the surrounding normal tissues (“Normal”). Meanwhile, quantified Western blotting assay results in Figure 1B confirmed Ppm1E protein upregulation in the gastric cancer tissues. Significantly, Ppm1E upregulation was correlated with AMPK in-activation (or AMPKα1 de-phosphorylation) and mTORC1 activation (or p-S6K1) in cancer tissues (Figure 1B). These results are not surprising, as Ppm1E is a defined AMPK phosphatase [33–35], and AMPK inhibition could lead to mTORC1 activation [19, 20, 36]. These results demonstrate Ppm1E upregulation in human gastric cancer tissues, which correlates with AMPK inhibition and mTORC1 activation.

### Ppm1E upregulation in human gastric cancer cells

**Figure 2:**
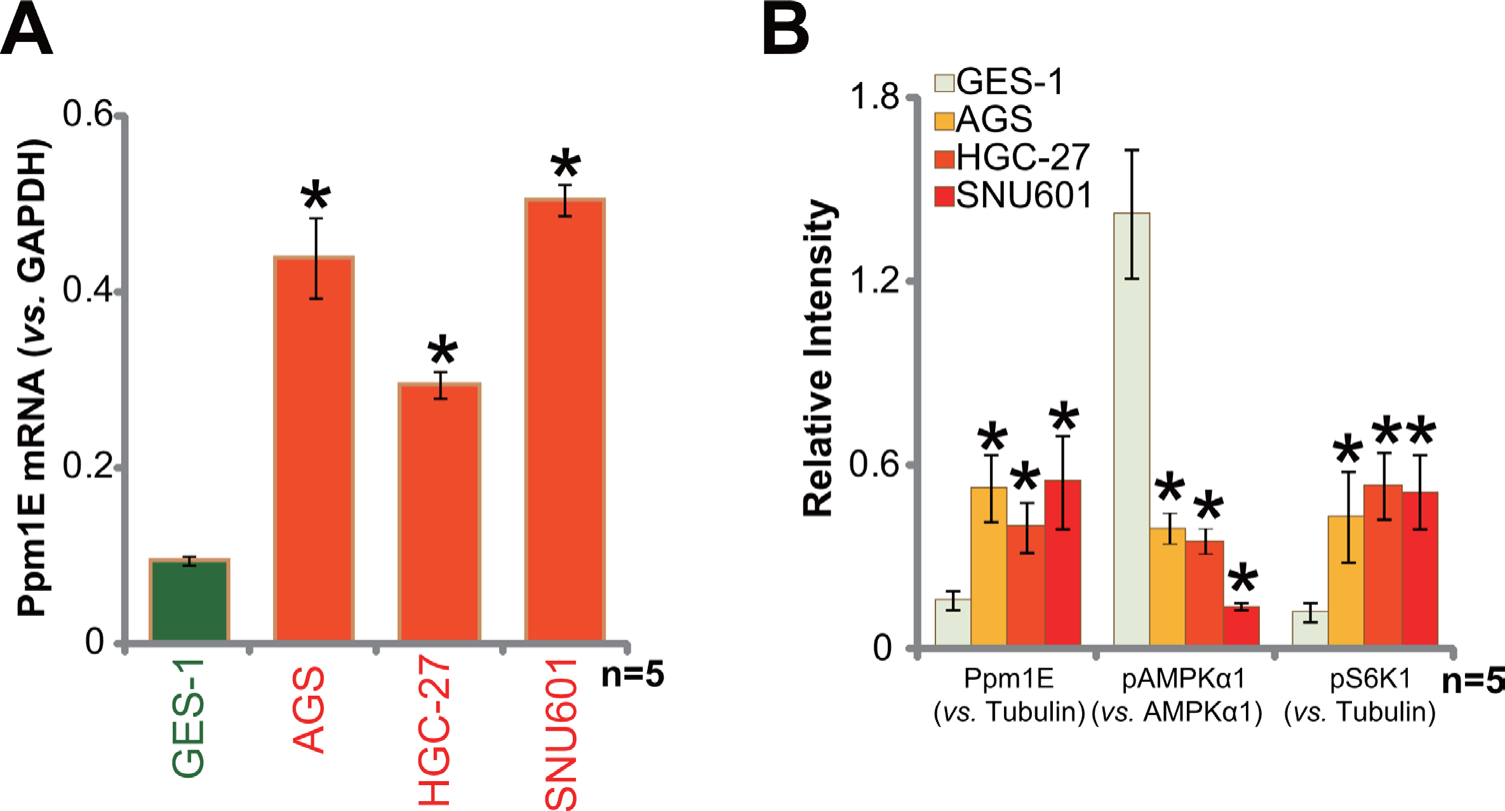
Ppm1E upregulation in human gastric cancer cells Human gastric cancer cell lines (AGS, HGC-27, and SNU601) and the gastric mucosal epithelial cell line GES-1 were subjected to qRT-PCR assay (**A**) and Western blotting assay (**B**) to test listed genes. Western blot data were quantified. *p < 0.05 *vs*. GES-1 cells. Experiments in this figure were repeated three times, and similar results were obtained.

We also tested Ppm1E expression in human gastric cancer cell lines. A total of three distinct gastric cancer cell lines, including AGS, HGC-27, and SNU601, were utilized. As compared to the GES-1 gastric mucosal epithelial cells, Ppm1E mRNA level was significantly higher in the above gastric cancer cells (Figure 2A). Ppm1E protein expression was also increased in above cancer cells (Figure 2B). Correspondingly, activation of AMPK, tested again by p-AMPKα1 at Thr-172, was decreased (Figure 2B), which was associated with mTORC1 activation (p-S6K1 increase, Figure 2B). These results demonstrate that the AMPK phosphotase is also upregulated in human gastric cancer cells, correlating with AMPKα dephosphorylation and mTORC1 activation.

### Ppm1E silence induces AMPK activation and inhibits gastric cancer cell survival and proliferation

**Figure 3:**
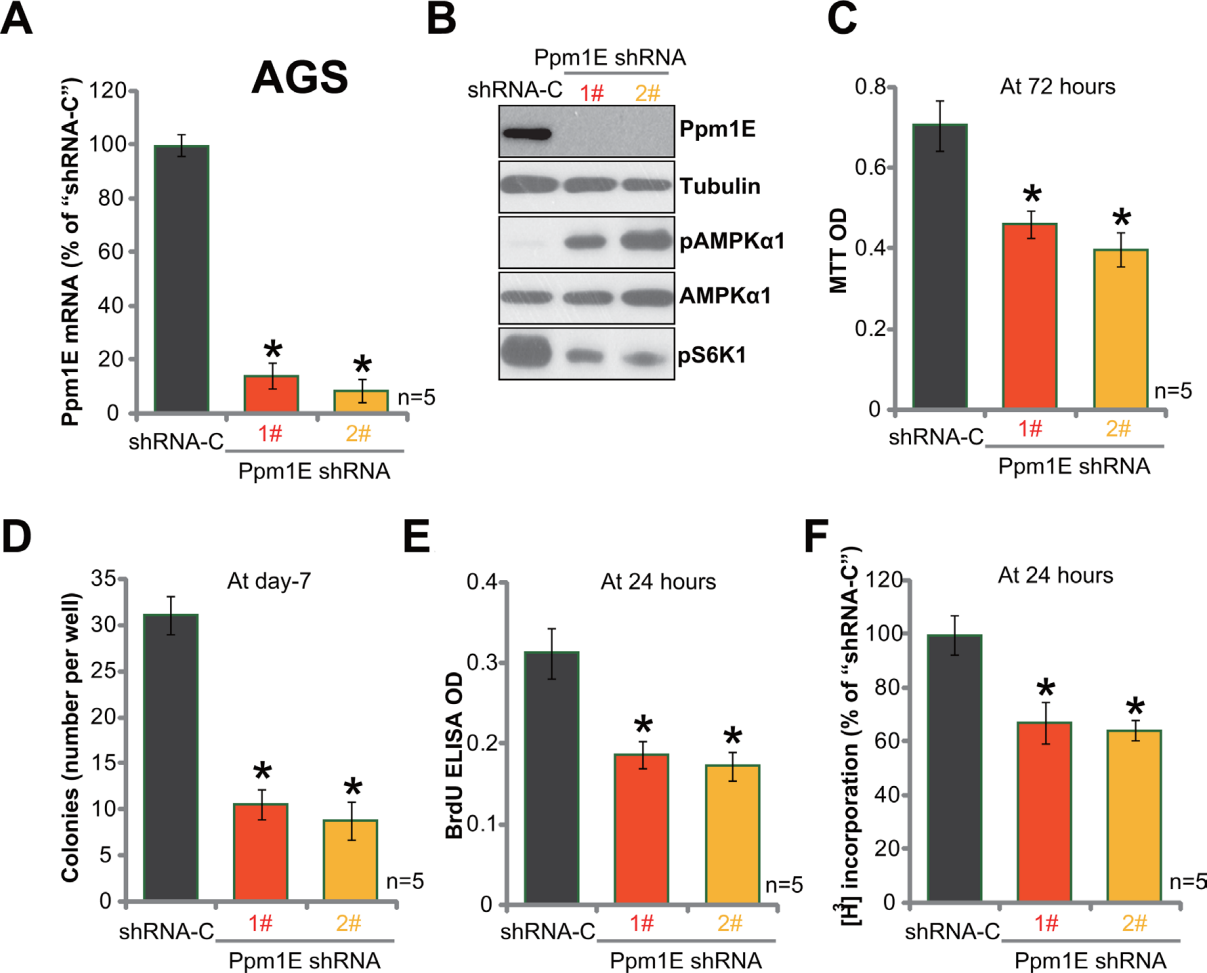
Ppm1E silence induces AMPK activation and inhibits gastric cancer cell survival and proliferation Expressions of Ppm1E mRNA (**A**) and listed proteins (**B**) in AGS cells with Ppm1E shRNA (“1#” or “2#”) or scramble control shRNA (“shRNA-C”) were shown. Above cells were also subjected to MTT assay (**C**), colony formation assay (**D**), BrdU ELISA assay (**E**) and DNA [H^3^] thymidine DNA incorporation assay (**F**) to test cell survival and proliferation. For these assays, exact same number of viable cells with listed shRNA was initially plated. *p < 0.05 *vs*. “shRNA-C” cells. Experiments in this figure were repeated four times, and similar results were obtained.

To study the possible function of Ppm1E in gastric cancer cell behaviors, shRNA strategy was utilized to knockdown Ppm1E in AGS cells. Two Ppm1E lentiviral shRNAs (“1#” and “2#”, gifts from Dr. Cui's group [35]), with non-overlapping sequences, were employed. qRT-PCR assay results in Figure 3A showed that the two shRNAs indeed potently downregulated Ppm1E mRNA in AGS gastric cancer cells. Further, Ppm1E protein expression was also depleted, which caused profound AMPKα1 phosphorylation (Figure 3B) and mTORC1 (p-S6K1) inhibition (Figure 3B). MTT assay results in Figure 3C showed that Ppm1E knockdown by shRNA decreased MTT viability optic density (OD) of AGS cells. Meanwhile, the number of survival AGS colonies was also decreased after expressing Ppm1E shRNA (Figure 3D). Cell proliferation was also tested by the BrdU ELISA assay and [H^3^] thymidine DNA incorporation assay. Results of both assays demonstrated that Ppm1E silence significantly inhibited AGS cell proliferation, as BrdU ELISA OD (Figure 3E) and [H^3^] thymidine DNA incorporation (Figure 3F) were both decreased after Ppm1E knockdown. These results clearly show that Ppm1E silence induces AMPK activation and inhibits survival and proliferation of AGS cells.

### Exogenous Ppm1E over-expression promotes gastric cancer cell survival and proliferation

**Figure 4:**
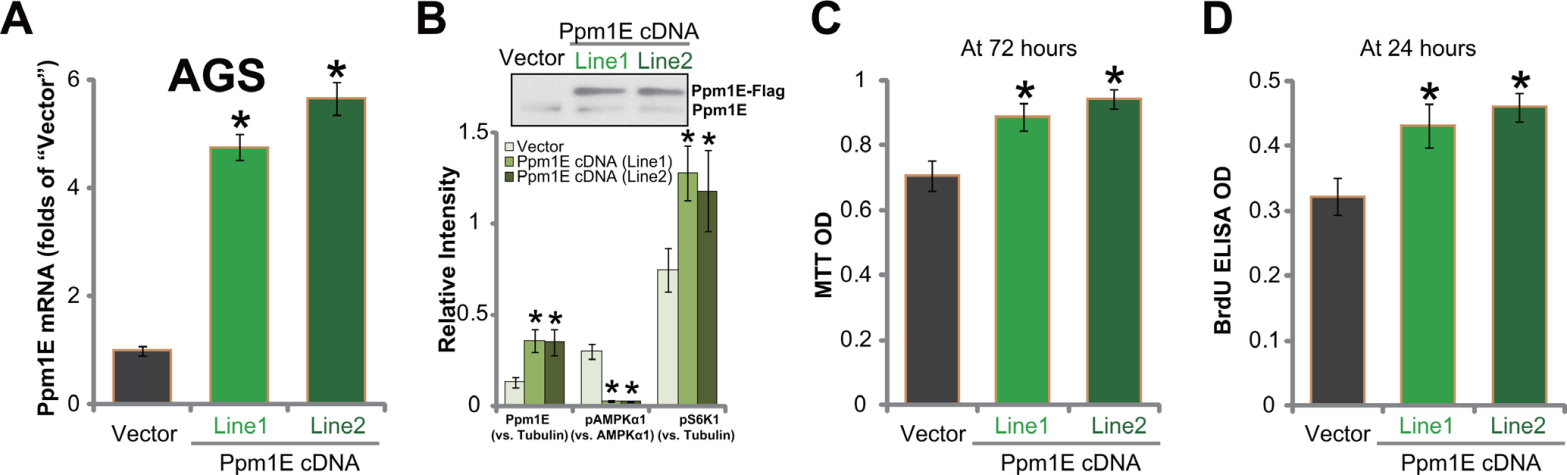
Exogenous Ppm1E over-expression promotes gastric cancer cell survival and proliferation Expressions of Ppm1E mRNA (**A**) and listed proteins (**B**, Data were quantified in the Lower panel) in stable AGS cell lines with exogenous Ppm1E (“Line1” and “Line2”, Flag-tagged) or empty vector (“Vector”) were shown. Above cells were also subjected to MTT assay (**C**) and BrdU ELISA assay (**D**) to test cell survival and proliferation, respectively. For these assays, exact same number of viable cells with listed shRNA was initially plated. *p < 0.05 *vs*. “Vector” cells. Experiments in this figure were repeated three times, and similar results were obtained.

To further confirm the function of Ppm1E in gastric cancer cell behaviors, we constructed the Ppm1E-expressing vector (see Methods). The construct was transfected to AGC cells. Through selection, two lines of AGS cells constitutively expressing the vector were established. They were named as “Line-1” and “Line-2”, respectively. Ppm1E mRNA was significantly upregulated in the two AGC cell lines (Figure 4A). Western blotting assay results in Figure 4B (Upper panel) further confirmed the exogenous Ppm1E expression (Flag-tagged) in the two lines. Notably, exogenous over-expression of Ppm1E led to further AMPKα dephosphorylation/inhibition (Figure 4B, Lower panel) and enhanced mTORC1 (p-S6K1) activation (Figure 4B, Lower panel). Remarkably, AGC cell viability (tested by MTT assay, Figure 4C) and proliferation (tested by BrdU ELISA assay, Figure 4D) were both augmented with exogenous Ppm1E expression. Therefore, Ppm1E over-expression facilitates mTORC1 activation and promotes gastric cancer cell survival and proliferation.

### Exogenous expression of miR-135b-5p causes Ppm1E depletion, AMPK activation, and proliferation inhibition in AGC cells

**Figure 5:**
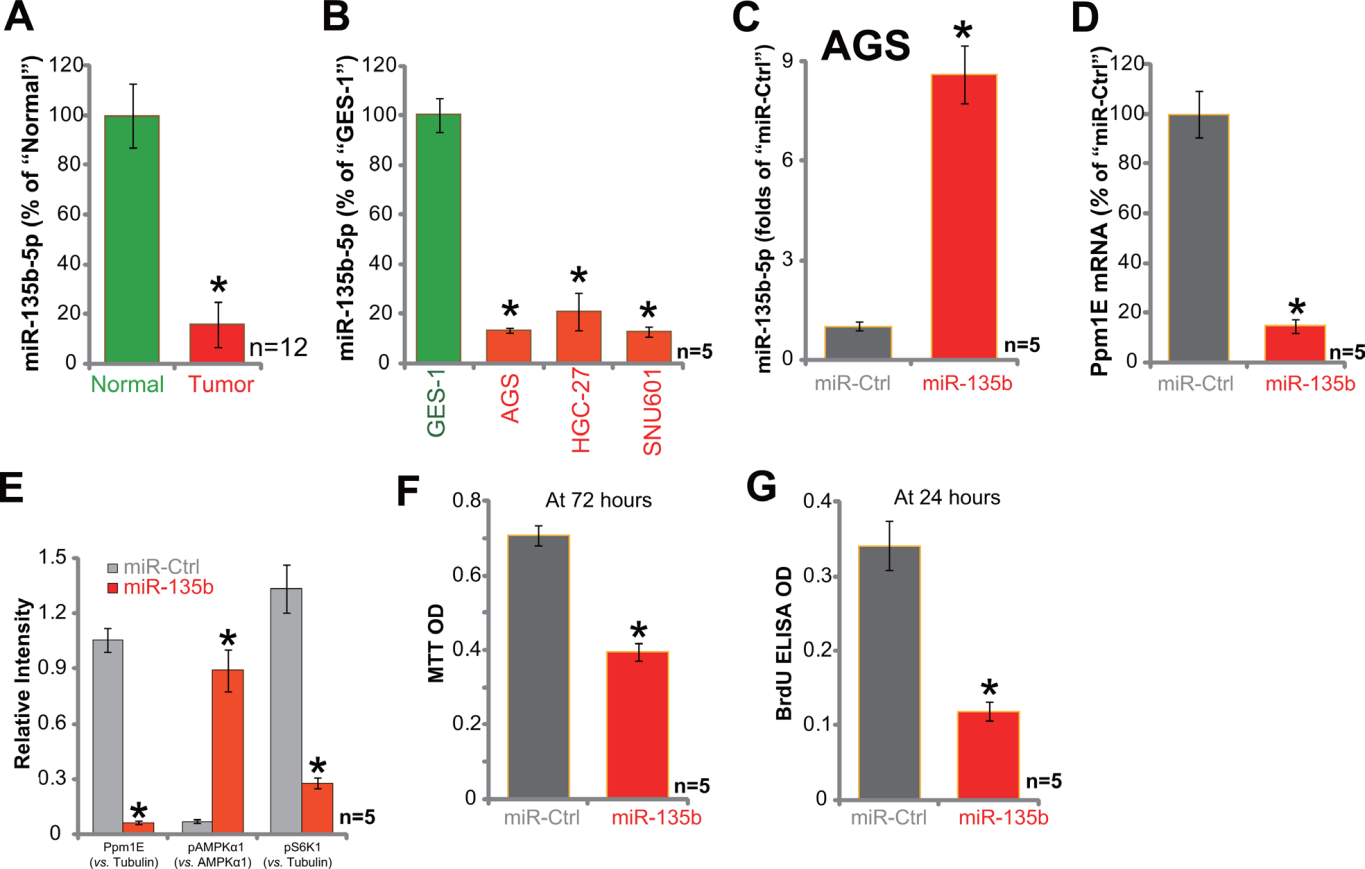
Exogenous expression of miR-135b-5p leads to Ppm1E depletion, AMPK activation, and proliferation inhibition in AGC cells The fresh human gastric cancer tissues (“Tumor”, *n* = 12) and the surrounding normal gastric tissues (“Normal”, *n* = 12), as well as gastric cancer cells (AGS, HGC-27, and SNU601) or GES-1 epithelial cells were subjected to qRT-PCR assay of microRNA-135b-5p (“miR-135b-5p”) expression (**A** and **B**). AGC cells, expressing miR-135b-vector or the miR-control vector (“miR-Ctrl”) were subjected to qRT-PCR assay testing expression of miR-135b-5p (**C**) and Ppm1E mRNA (**D**) Listed proteins were also tested by Western blotting assay, and blot data were quantiifed (**E**) Cells were also subjected to MTT assay (**F**) and BrdU ELISA assay (**G**). For these assays, exact same number of viable cells with listed vector was initially plated. *p < 0.05 *vs*. “Normal” tissues (A) or GES-1 cells (B). *p < 0.05 *vs*. “miR-Ctrl” cells (C–G). Experiments in this figure were repeated three times, and similar results were obtained.

Next, we focused on the possible cause of Ppm1E upregulation in gastric cancer tissues and cells. Several very recent studies have characterized a Ppm1E-targeting miRNA: namely microRNA-135b-5p (“miR-135b-5p”) [34, 35]. We therefore tested expression of this miRNA in above tissues and cells. Remarkably, as shown in Figure 5A, miR-135b-5p level was dramatically downregulated in human gastric cancer tissues. Meanwhile, its level was also quite low in the tested gastric cancer cell lines (Figure 5B). Next, a miR-135b-expressing vector (a gift from Dr. Cui [35, 37]) was introduced to AGS cells. qRT-PCR assay results showed that AGS cells with miR-135b vector showed significantly increased miR-135b-5p expression (Figure 5C). Reversely, *Ppm1E* mRNA (Figure 5D) and protein (Figure 5E) was depleted. Forced miR-135b-5p expression similarly induced AMPK activation (p-AMPKα1, Thr-172) and mTORC1 (p-S6K1) inhibition (Figure 5E, results were quantified). Meanwhile, miR-135b-5p expression also inhibited AGC cell survival and proliferation, which were again tested by the MTT assay (Figure 5F) and BrdU ELISA assay (Figure 5G), respectively. Together, we show that exogenous expression of miR-135b-5p causes Ppm1E depletion, AMPK activation, and proliferation inhibition in AGC cells.

## DISCUSSION

The possible AMPKα phosphatases are largely unknown until recently. A group of studies have implied Ppm1E as a vital AMPKα phosphatase [33–35]. On the other hand, genetic inhibition of Ppm1E could provoke AMPKα1 phosphorylation or AMPK activation [33–35]. Here, we showed that Ppm1E was significantly upregulated in both human gastric cancer tissues and gastric cancer cell lines, which was correlated with AMPKα dephosphorylation/inhibition. Ppm1E knockdown by shRNA then activated AMPK and significantly inhibited human gastric cancer proliferation. Reversely, forced over-expression of Ppm1E induced further AMPK inhibition to promote gastric cancer cell proliferation. Therefore, Ppm1E, the AMPKα phosphatase, has the potential to be a novel and valuable oncotarget protein for human gastric cancer.

Activation of mTORC1 participates in almost all cancerous behaviors [3, 38, 39]. mTORC1 is often dysregulated and constitutively-activated in human gastric cancers, representing a key oncotarget for treatment [3]. Numerous studies, including ours [11–15, 40], have demonstrated that AMPK activation could inhibit mTORC1. There are two following mechanisms for mTORC1 inhibition by AMPK. Activated AMPK indirectly inhibits mTORC1 via phosphorylating and activating TSC2, the latter is a known mTORC1 upstream inhibitor protein (TSC2-dependent) [19]. Further, AMPK could also phosphorylate and in-activate mTORC1 component Raptor to directly inhibit mTORC1 (TSC2-independent) [41, 42]. In this study, we showed that Ppm1E knockdown by shRNA activated AMPK in gastric cancer cells, which led to mTORC1 in-activation and proliferation inhibition. Following studies will be needed to confirm that mTORC1 inhibition is the reason of gastric cancer cell proliferation inhibition by Ppm1E shRNA.

Very recent studies have characterized miR-135b-5p as the Ppm1E-targeting miRNA. Here we found that miR-135b-5p was significantly downregulated in human gastric cancer tissues and cell lines. Remarkably, exogenous expression of miR-135b-5p induced Ppm1E downregulation, AMPK activation, and proliferation inhibition in AGC cells. Therefore, miR-135b-5p depletion could be the cause of Ppm1E upregulation in human gastric cancer tissues/cells. The detailed underlying mechanisms warrant further investigations.

## MATERIALS AND METHODS

### Culture of established cell lines

Human gastric cancer cell lines, AGS, HGC-27, and SNU601, as well as human gastric mucosal epithelial cell line GES-1 were purchased from the Cell Bank of CAS Shanghai (Shanghai, China) at Dec 2013. Cells were maintained in RPMI medium with 10% fetal bovine serum (FBS). The cell line verification was described previously [43].

### Reagents and antibodies

Puromycin was purchased from Sigma (Shanghai, China). The Ppm1E antibody was from Dr. Cui's group [35]. All other antibodies utilized in this study were obtained from Cell Signaling Tech (Danvers, MA). The cell culture reagents were obtained from Gibco Life Technologies (Carlsbad, CA).

### Isolation of human gastric cancer tissues

Surgery-isolated primary human gastric cancer tissue specimens were washed in DMEM. Tumor tissues and surrounding normal tissues were separated under microscopy very carefully. A total of twelve (12) different patients with primary gastric cancer, administered at authors’ institution, were enrolled (8 male, 4 female, 42–67 years old). Enrolled patients received no chemotherapy or radiotherapy prior to surgery. Fresh tissues were stored in liquid nitrogen. Tissue lysis buffer (Biyuntian, Wuxi, China) was applied to homogenate the tissue samples [44–47]. Experiments and protocols requiring human samples were approved by the Internal Review Board (IRB) of all authors’ institutions. The written-informed consent was obtained from each participant. All studies using human samples were conducted according to the principles expressed in the Declaration of Helsinki and national/international guidelines.

### RNA extraction and real-time PCR

As previously reported [13, 43, 48], total RNA from fresh cellular and tissue lysates was prepared via the TRIzol reagents (Invitrogen). Quantitative Real Time-PCR (“qRT-PCR”) assay was performed. The PCR reaction mixture had SYBR Master Mix (Applied Biosystem), 0.5 μg RNA and 100 nM primers. The ABI Prism 7500 Fast Real-Time PCR system (Foster City, CA) was employed for PCR reactions. The mRNA primers for Ppm1E and GAPDH were provided by Dr. Cui's group [35, 37]. The primers for miR-135b-5p were also gifts from Dr. Cui [35, 37]. Melt curve analysis was tested to analyze product melting temperature. GAPDH was always analyzed as the reference gene. The 2^−ΔΔ*C*t^ method was applied to quantify targeted gene/miRNA expression change within samples [13, 48].

### Western blotting assay

As described previously [43, 46, 49, 50], aliquots of 30 μg of lysate proteins from cell or tissue samples were separated by SDS-page gel (10–12%), and were transferred onto polyvinylidene difluoride (PVDF) membranes (Millipore, Shanghai, China). After blocking, membranes were added with specific primary and corresponding secondary antibodies. Enhanced chemiluminescence (ECL) reagents (Amersham Bioscience, Freiburg, Germany) were applied for detection the interested band. The intensity of each band was quantified by ImageJ software.

### MTT assay

Cell viability/survival was tested by the 3-(4,5-dimethylthiazol-2-yl)-2,5-diphenyltetrazolium bromide (MTT) assay as described [15].

### [H^3^] Thymidine incorporation assay of cell proliferation

As descried [43, 51], cells with indicated treatment was incubated with 1 μCi/ml of tritiated thymidine (Sigma, China). To determine [H^3^] thymidine incorporation, cells were washed with PBS. Afterwards, cold 10% trichloroacetic acid (TCA) was applied to precipitate DNA, which was then solubilized with 1.0 M sodium hydroxide. The aliquots were counted by liquid-scintillation spectrometry [43, 51].

### BrdU ELISA assay of cell proliferation

Cells with different genetic modifications were incubated with BrdU (10 μM, Cell Signaling Tech, Shanghai, China). BrdU incorporation was determined in the ELISA format [47].

### Colony formation assay

Colony formation was also performed to test cell proliferation. Briefly, cells (1 × 10^4^) with different genetic modification were seeded onto the 6-well tissue culture plate, which were allowed to attach for 24 hours. Cells were further cultured for additional 7 days. Afterwards, colonies were stained with crystal violet solution and counted.

### Ppm1E knockdown by shRNA

Two non-overlapping Ppm1E lentiviral shRNA plasmids were provided by Dr. Cui [35]. These two Ppm1E shRNAs were added to cultured AGS cells (with 60% confluence) directly. After 24 hours, virus-containing medium was replaced with fresh complete medium. Stable AGS colonies were then selected by puromycin (2.5 μg/mL, Sigma) for 4 days. Expression of Ppm1E in the stable cells was tested by Western blotting assay.

### Exogenous expression of Ppm1E

A full-length Ppm1E cDNA (provided by Genepharm, Kunshan, China) was sub-cloned into the pSV2 neo Flag plasmid [43], which was transfected into AGS cells by Lipofectamine 2000 (Invitrogen) reagents. After 24 hours, cells were re-plated on selection medium containing 100 μg/mL of G418 for 4 days. Expression of Ppm1E (Flag-tagged) in the resulting cells was tested by qRT-PCR assay and Western blotting assay.

### microRNA-135b (miRNA-135b) transfection

miRNA-135b expression vector and non-sense miRNA-control (“miR-Ctrl”) vector were gifts from Dr. Cui [35, 37]. Cells were seeded on to six-well plates with 60% confluence. Lipofectamine 2000 transfection reagent (Invitrogen) was utilized for transfection the construct (0.15 μg construct per transfection). After 24 hours, cell medium was replaced with 2 mL of complete medium. Puromycin (2.0 μg/mL, Sigma) was then added to establish stable cells for 4 days. Expression of miRNA-135b-5p and Ppm1E in above cells was always tested.

### Statistical analysis

Data were presented as mean ± standard deviation (SD). Statistics were analyzed by one-way ANOVA followed by a Scheffe’ and Tukey Test (SPSS 16.0, Chicago, CA). *p* < 0.05 means significant difference.

## CONCLUSIONS

In summary, we show that Ppm1E is upregulated in both human gastric cancer tissues/cell lines, which apparently is important for shutting down AMPK signaling and promoting cancer cell proliferation.
